# TCVS: tree-guided compositional variable selection analysis of microbiome data

**DOI:** 10.1093/bioinformatics/btaf617

**Published:** 2025-11-09

**Authors:** Yicong Mao, Zhiwen Jiang, Tianying Wang, Yijuan Hu, Xiang Zhan

**Affiliations:** Department of Biostatistics, School of Public Health, Peking University, Beijing, 100191, China; Department of Biostatistics, University of North Carolina, Chapel Hill, Dauer, NC 27599, United States; Department of Statistics, Colorado State University, Fort Collins, CO 80523, United States; Department of Biostatistics, School of Public Health, Peking University, Beijing, 100191, China; Beijing International Center for Mathematical Research and Center for Statistical Science, Peking University, Beijing, 100871, China; School of Statistics and Data Science, Southeast University, Nanjing, 211189, China

## Abstract

**Motivation:**

Studies of microbial communities, represented by the relative abundances of taxa at various taxonomic levels, have underscored the significance of microbiota in numerous aspects of human health and disease. A pivotal challenge in microbiome research lies in pinpointing microbial taxa associated with disease outcomes, which could play crucial roles in prevention, detection, and treatment of various health conditions. Alongside these relative abundance data, taxonomic information sometimes offers a unique lens to explore the impact of shared evolutionary histories on patterns of microbial abundance.

**Results:**

In pursuit of this goal, we utilize the tree structure to more flexibly identify taxa associated with disease outcomes. To enhance the accuracy of our selection process, we introduce auxiliary knockoff copies of microbiome features designated as noise. This approach allows for the assessment of false positives in the selection process and aids in refining it towards more precise outcomes. Extensive numerical simulations demonstrate that our methodology outperforms several existing methods in terms of selection accuracy. Furthermore, we demonstrate the practicality of our approach by applying it to a widely used gut microbiome dataset, identifying microbial taxa linked to body mass index.

**Availability and implementation:**

TCVS R code is available at https://github.com/Yicong1225/TCVS.

## 1 Introduction

Among the array of microbiome studies, marker gene sequencing, particularly of the 16S rRNA gene, emerges as a prevalent high-throughput technique for profiling microbial communities (Hamady and Knight 2009). This process typically involves clustering raw 16S sequencing reads into Operational Taxonomic Units (OTUs) based on sequence similarity. An OTU’s abundance is determined by the total count of sequence reads aligned to it, forming an OTU by sample abundance table—known as the OTU table—which serves as a foundation for subsequent statistical analyses (Li 2019). Despite advancements in sequencing techniques in microbiome research, the development of statistical methods for the effective and robust analysis of microbiome data lags behind, which is probably due to certain unique data characteristics that are described as follows.

The inherent compositionality of next-generation microbiome sequencing data presents a primary challenge in data analysis. These data inherently provide information in relative abundances due to the loss of total abundance information during the sequencing process, reflecting compositional information of unique sequences (Gloor *et al.* 2016, Vandeputte *et al.* 2017). Additionally, the normalization of OTU counts into proportions, to mitigate vast discrepancies in sequencing depths, further emphasizing the compositional structure of microbiome data (Morton *et al.* 2019). The statistical community has responded by proposing a variety of tools for analyzing compositional microbiome data within a simplex space, facilitating rigorous statistical inference (Lin *et al.* 2014, Shi *et al.* 2016, Sohn *et al.* 2019, Srinivasan *et al.* 2021, Hu *et al.* 2022, Li *et al.* 2024, Rios *et al.* 2025). Beyond compositionality, microbiome data analysis is further complicated by the “small n large p” problem. Typical microbiome studies feature relatively small sample sizes, especially when compared to genetic studies, which limits the applicability of traditional asymptotic-based methods for analysis of microbiome data. For example, in association analysis, methods designed for large-sample genetic studies have been shown to yield conservative outcomes in microbiome contexts (Zhan *et al.* 2017). In the realm of statistical variable selection, previous studies have indicated a tendency towards excessive false positives in analyses of small-sample microbiome data (Susin *et al.* 2020, Srinivasan *et al.* 2021). Moreover, a third challenge for effective analysis of microbiome data is accommodating complex data structures (Jiang *et al.* 2021, 2023). For example, a pivotal technique in microbiome research is 16S rRNA gene sequencing, which utilizes the 16S rRNA gene as a consistent phylogenetic marker to explore the lineage relationships among microbial taxa (Hamady and Knight 2009). These OTUs are further categorized into a hierarchical taxonomy—spanning from species to kingdom—reflecting evolutionary connections. Proper exploitation of this taxonomic architecture can enhance both biological relevance and statistical robustness of various tree-based approaches (Chen *et al.* 2012, Shi and Li 2017, Tang *et al.* 2017, Wang *et al.* 2017, Tang *et al.* 2018, Bogomolov *et al.* 2021, Li *et al.* 2022, Mao and Ma 2022, Shi *et al.* 2023). A foundational premise of these tree-based methods is the assumption that OTUs with closer taxonomic relationships are likely to exhibit more similar biological functions (Hendry *et al.* 2011).

Our primary objective is to identify microbial taxa linked with disease outcomes, aiming to inform and improve medical decision-making related to the design of microbiome-based interventions for the prognosis, diagnosis, and treatment of various diseases. In light of the analytical challenges discussed previously, our approach is specifically designed to address the issue of compositionality in variable selection analysis and seeks to integrate taxonomic structure information, thereby increasing the biological relevance and statistical efficacy of our findings. Traditional approaches for taxa selection typically employ the multiple testing framework, which analyze each taxon separately and subsequently apply corrections for multiple comparisons (Wang and Jia 2016). However, recent findings suggest that marginal analysis of individual taxa, which overlook negative correlations among taxa due to compositionality, may yield inaccurate interpretations (Hawinkel *et al.* 2019). Thus, new methods are desired.

A conventional method for identifying disease-associated microbial taxa involves performing statistical variable selection analysis (Lin *et al.* 2014). However, this approach tends to yield a high false positive rate (Susin *et al.* 2020). To improve selection accuracy, one promising strategy is to introduce auxiliary variables that are likely to be identified as false positives. This approach facilitates the evaluation of selection outcomes, encouraging a selection process that favors true variables over these artificially introduced noises. Notable examples of such auxiliary noises include the knockoff copies (Barber and Candès 2015, Candes *et al.* 2018) and permutations (Wu *et al.* 2007, Yang *et al.* 2020). A significant challenge remains in effectively incorporating taxonomic tree information to enhance both biological interpretability and statistical power. Previous efforts have either focused on developing new tree-based penalties to encourage sparsity (Wang *et al.* 2017, Liu *et al.* 2022) or on defining novel error rates for error-controlled hypothesis testing or taxa selection (Bogomolov *et al.* 2021, Li *et al.* 2022). Our proposed method, Tree-guided Compositional Variable Selection (TCVS), diverges from these approaches by utilizing tree structure to facilitate group variable selection.

The remaining of this article is organized as follows. Section 2 first provides essential background on regression with compositional covariates and taxonomic tree within microbiome data, and then introduces the new TCVS method. In Section 3, we demonstrate TCVS’s numerical properties via simulation studies, followed by its application to a real-world microbiome dataset. We conclude with a brief discussion in Section 4.

## 2 Materials and methods

### 2.1 Notation and preliminaries

Consider a compositional vector $x^{⊤}=(x_{1},\ldots ,x_{p})$ situated within simplex $S^{p}=\{(x_{1},\ldots ,x_{p}):\sum_{j=1}^{p}x_{j}=1,x_{j}>0,j=1,\ldots ,p\}$. The literature on regression analysis of compositional data frequently employs log-contrast models using log-ratio transformations of **x** as predictors (Aitchison 1982, Aitchison and Bacon-Shone 1984). Two famous examples are the log-contrast model with Additive Log-Ratio (ALR) transformation:


(1) $$y=\delta_{0}+\sum_{j=1}^{p}\delta_{j}\;\text{log}(x_{j})+\epsilon ,\;s.t.,\sum_{j=1}^{p}\delta_{j}=0,$$

and with Centered Log-Ratio (CLR) transformation:


(2) $$y=\beta_{0}+\sum_{j=1}^{p}\beta_{j}\;\text{log}(x_{j}^{c})+\epsilon ,\;s.t.,\sum_{j=1}^{p}\beta_{j}=0,$$

where $x_{j}^{c}=x_{j}/{\bar{x}}^{g}$ and ${\bar{x}}^{g}=\sqrt[{p}]{x_{1}\cdots x_{p}}$ represents the geometric mean of **x**. Both models have been widely used (Lin *et al.* 2014, Wang *et al.* 2017), and a subtle difference between two models lies in the CLR model’s efficacy in approximating compositional data to a multivariate normal distribution, as evidenced in prior studies (Shi *et al.* 2016, Wang *et al.* 2017). This characteristic of the CLR transformation is particularly advantageous for the objective of this paper, as it facilitates creation of effective auxiliary noises. Hence, the development of our methodology will predominantly leverage the CLR model framework (2) moving forward.

Statistical analysis of microbiome data often extends beyond the OTU abundance table to include a taxonomy table that details the hierarchical classification of each OTU. The taxonomic structure implied in the taxonomy table can often be visualized using a taxonomic tree, displaying the lineage of each OTU as leaf nodes and higher taxonomic ranks (e.g., kingdom, phylum, class, order, family, genus) as internal nodes. The integration of such a taxonomic tree structure into statistical analyses of microbiome data has garnered significant interest and recent advancements in this area have spanned a broad spectrum of applications, including association analysis (Chen *et al.* 2012, Tang *et al.* 2017, Wang and Zhao 2017), hypothesis testing (Shi and Li 2017, Tang *et al.* 2018, Bogomolov *et al.* 2021, Li *et al.* 2022), clustering (Mao and Ma 2022), mediation analysis (Hong *et al.* 2023). One notable innovation within the aforementioned vein is the TASSO method (Wang *et al.* 2017), which introduces a tree-based penalty to enhance sparsity in variable selection. However, TASSO predominantly promotes sparsity among subcompositions—internal nodes of the tree—through linear contrasts of leaf nodes. This approach overlooks the practical need for direct intervention at the OTU level, as altering the abundance of specific taxa is often more feasible and relevant for disease treatment than modifying a group of taxa. Recognizing this gap, our method aims to achieve selection sparsity directly among leaf nodes of the tree.

### 2.2 Tree-guided compositional variable selection (TCVS)

Existing methods for variable selection analysis of microbiome compositional data predominantly employ lasso-type approaches to identify microbial features associated with disease outcomes (Lin *et al.* 2014, Wang *et al.* 2017). However, empirical research has revealed that the compositional lasso-based techniques may lead to excessive false positives—a situation exacerbated by the small sample sizes in microbiome research (Susin *et al.* 2020, Srinivasan *et al.* 2021). The identification of such false positives not only misguides the interpretation of results but also may result in the allocation of substantial resources towards unproductive validation and functional exploration efforts. Thus, new methods capable of delivering more precise selection outcomes are needed.

To enhance the precision of variable selection, a prevalent approach involves the integration of auxiliary noise variables or pseudo-variables into an augmented regression model. Notable implementations of such auxiliary noises are knockoffs and permutations (Barber and Candès 2015, Candes *et al.* 2018, Wu *et al.* 2007, Yang *et al.* 2020). The underlying principle of incorporating auxiliary noises stems from the challenge of distinguishing inactive variables from active ones within the original dataset. By introducing auxiliary noises that simulate the behavior of original variables in an augmented regression model, it becomes feasible to assess the relative significance of each variable against its artificial counterpart. Essentially, this process combats the inherent noise within the original variables by employing auxiliary noises, thereby marking the less significant ones as clear false positives. The knockoff filter (KF) framework stands out as a prominent example within this context, renowned for its ability to ensure false discovery rate-controlled variable selection (Barber and Candès 2015, Candes *et al.* 2018). Owing to its effectiveness and widespread recognition, we choose to implement knockoffs as the auxiliary noises to develop TCVS in this article.

The fixed-X knockoff necessitates a Gaussian outcome and a sample size surpassing the number of variables (Barber and Candès 2015). Conversely, the model-X knockoff mandates a comprehensive understanding of the covariates’ joint distribution for building knockoff features (Candes *et al.* 2018). Srinivasan *et al.* (2021) adapted the ALR model to propose the compositional knockoff filter (CKF), initiating with a screening phase for dimensionality reduction on compositional covariates, followed by employing the fixed-X knockoff for microbiome taxa selection from the residual data. However, integrating taxonomic tree information into this bifurcated CKF process poses significant challenges. The initial dimensionality reduction necessitates the pruning of the tree into a subtree, where different divisions of the sample could result in variant subtrees, complicating the interpretability of subsequent tree-based variable selection stages. Alternatively, employing the model-X knockoff framework requires prior knowledge of predictor distributions to formulate knockoff features. Given these considerations, we pivot towards utilizing the CLR model for developing our TCVS approach. This decision is underpinned by evidence in previous literature demonstrating the CLR model’s efficacy in approximating compositional data to a multivariate normal distribution (Shi *et al.* 2016, Wang *et al.* 2017). In addition, we empirically verified this multivariate normality assumption in both our simulation studies and real data analyses. Further details are provided in Section B.7 of the online supplementary materials.

Let T denote the taxonomic tree as a hierarchical representation of the taxonomy structure of *p* OTUs identified in the study. The tree’s nodes are bifurcated into two categories: *p* leaf nodes L, each symbolizing an OTU, and internal nodes I, each representing taxa at a higher taxonomic rank than that of an OTU. For simplicity, we denote leaf nodes as L={1,2,…,p}. For any internal node *v* within I, $T_{v}$ signifies the subtree with *v* as the root, and $L_{v}$ represents the subset of leaf nodes within this subtree. Accompanying each internal node *v* is a *p*-dimensional membership indicator vector $m_{v}={\left(m_{1},\ldots ,m_{p}\right)}^{⊤}$, with $m_{j}=1$ if leaf node *j* belongs to $L_{v}$ and 0 otherwise. Assuming **X** as the matrix of *n* samples with *p* compositional components and **y** as the response vector dependent on **X** or CLRs $Z={\left\{Z_{ij}\right\}}_{n\times p}=\log(x_{ij}/{\bar{x}}_{i}^{g})$. Leveraging the tree structure, we propose a group lasso-type penalty to facilitate variable selection, as described by the following equation:


(3) $$\begin{array}{cc} \hat{\beta } & =\text{arg}\;\text{min}\beta \{\frac{1}{2n}∥y-Z\beta {∥}_{2}^{2}\\ & +\lambda \left(∥\beta {∥}_{1}+\sum_{v\in I}\frac{∥\beta_{m_{v}}{∥}_{2}}{∥m_{v}{∥}_{1}}\right)\},\;s.t.\;\;\sum_{j=1}^{p}\beta_{j}=0, \end{array}$$

where $\beta ={\left(\beta_{1},\ldots ,\beta_{p}\right)}^{⊤}$ represents the regression coefficients, $||\beta |{|}_{1}$ and $||\beta |{|}_{2}$ denote the L_1_ and L_2_ norms of β respectively, $\beta_{m_{v}}$ is the element-wise multiplication of the membership vector $m_{v}$ and β, and $||m_{v}|{|}_{1}$ denotes the number of leaf nodes of the internal node. This model innovates over the compositional lasso method by incorporating an additional penalty term $\sum_{v\in I}||\beta_{m_{v}}|{|}_{2}$, leveraging the tree structure for enhanced variable selection. Also the denominator $||m_{v}|{|}_{1}$ of the group penalty term takes the size of each internal node into account to offset potential adverse effects of a very unbalanced tree topology. In contrast to TASSO (Wang *et al.* 2017), which assigns a coefficient sum for each internal node *v* and tackles a generalized lasso problem with both leaf and internal nodes as predictors, our TCVS approach focuses solely on leaf nodes. TCVS is rooted in the biological premise that evolutionarily related species often respond to external perturbations in a similar manner.

To enhance selection accuracy of the estimator delineated in Equation (3), auxiliary noises, specifically designed knockoff copies $\tilde{Z}=({\tilde{Z}}_{1},\ldots ,{\tilde{Z}}_{p})$ of **Z**, are utilized. These copies are generated by the sequential conditional independent pairs algorithm (Candes *et al.* 2018) to **Z**. By design, variables $\tilde{Z}$ hold no association with the outcome **y**, thus acting as control variables against the original set **Z**. Importantly, the creation of knockoff copies $\left({\tilde{Z}}_{1},\ldots ,{\tilde{Z}}_{p}\right)$ for the original variables $\left(Z_{1},\ldots ,Z_{p}\right)$ preserves the existing tree structure. Consequently, the augmented tree comprises the same number of internal nodes, denoted as |I|, but now includes 2*p* leaf nodes. This necessitates a redefinition of the leaf node membership vector to $M_{v}={\left(m_{1},\ldots ,m_{2p}\right)}^{⊤}$, where $m_{j}$ retains its original definition for j=1,…,p, and for j=(p+1),…,2p, we set $m_{j}=m_{j-p}$. The augmented problem incorporating the design matrix $\left(Z,\tilde{Z}\right)$ is now formalized as:


(4) $$\begin{array}{c} (\hat{\beta },\hat{\tilde{\beta }})=\underset{(\beta ,\tilde{\beta })\in F}{\text{arg}\;\text{min}\;}[\frac{1}{2n}||y-Z\beta -\tilde{Z}\tilde{\beta }|{|}_{2}^{2}+\lambda \{||\beta |{|}_{1}\\ +||\tilde{\beta }|{|}_{1}+\sum_{v\in I}\frac{||\beta_{M_{v}}^{*}|{|}_{2}}{||M_{v}|{|}_{1}}\}], \end{array}$$

where $F=\left\{(\beta ,\tilde{\beta })|\sum_{j=1}^{p}\beta_{j}=0,\sum_{j=1}^{p}{\tilde{\beta }}_{j}=0\right\}$ is the feasible set and $\beta_{M_{v}}^{*}$ is the element-wise product of vector $M_{v}$, $\beta^{*}={\left(\beta^{⊤},{\tilde{\beta }}^{⊤}\right)}^{⊤}$, and $||M_{v}|{|}_{1}$ denotes the number of 1’s in the membership vector $M_{v}$.

Following the knockoff method (Candes *et al.* 2018), we determine the relative importance of each original variable $Z_{j}$ in comparison to its knockoff counterpart ${\tilde{Z}}_{j}$ by computing:


(5) $$W_{j}=|\beta_{j}|-|{\tilde{\beta }}_{j}|,$$

where a significantly positive $W_{j}$ suggests a higher likelihood of $Z_{j}$ being an influential predictor. To ascertain the threshold value for the significance metrics, the methodology adheres to the KF approach by employing the threshold criterion defined as:


(6) $$T=\min\left\{t\in W:\frac{\#\{j:W_{j}\leq -t\}}{1\vee \#\{j:W_{j}\geq t\}}\leq q\right\},$$

where $W=\{|W_{j}|:j=1,2,\ldots ,p\}\setminus \{0\}$ represents the set of distinct, non-zero absolute values of $W_{j}$, a∨b signifies the maximum between *a* and *b*, and q∈(0,1) is the predefined cutoff level chosen by the user. Consequently, the proposed TCVS scheme identifies the set of pertinent variables as $\hat{S}=\{j:W_{j}\geq T\}$.

For simplification purposes, this exposition omits the discussion on other potential covariates (e.g., age and gender) within the TCVS framework, highlighting that the methodology can be seamlessly extended to incorporate additional covariates alongside the microbiome variables **Z** and $\tilde{Z}$ as delineated in problems (3) and (4). The comprehensive procedure of our method is succinctly encapsulated in Algorithm 1 for thorough understanding.

Algorithm 1 Tree-guided Compositional Variable Selection (TCVS)

**Input:** Centered log-ratio matrix Z, response Y, taxonomic tree structure T, threshold q∈(0,1)

**Output:** A selection set $\hat{S}$

**Procedure:** 

1. Generate knockoff copies $\tilde{Z}$ of Z;

2. Solve the augmented regression problem (4);

3. Calculate the absolute coefficient differences $W_{j}^{\prime }s$ in (5) and then the cutoff value *T* in (6) for a user pre-specified *q* value;

4. Obtain the selected set as $\hat{S}=\{j:W_{j}\geq T\}$.

Finally, the TCVS algorithm involves selection of tuning parameters including λ and *q*. The parameter λ is instrumental in balancing the model’s goodness-of-fit and parsimony and this balance is typically achieved through methods such as cross-validation or various information criteria. In this context, we employ the Bayesian Information Criterion (BIC) (Schwarz 1978) to select the optimal tuning parameter λ and the selection process involves minimizing the following function:


$$\mathrm{BIC}(\lambda )=n\;\text{log}\;\left[\frac{∥y-Z^{*}{\hat{\beta }}^{*}{∥}_{2}^{2}}{n}\right]+\hat{K}(\lambda )\;\text{log}\;n,$$

where $Z^{*}=(Z,\tilde{Z})$ denotes the augmented design matrix, $\beta^{*}={\left(\beta^{⊤},{\tilde{\beta }}^{⊤}\right)}^{⊤}$ represents the combined parameter vector, and $\hat{K}(\lambda )=\{j:{\hat{\beta }}_{j}^{*}(\lambda )\neq 0\}$ signifies the count of non-zero coefficients estimated under λ. The optimal tuning parameter, $\lambda^{\text{opt}}$, is identified as the one yielding the minimum BIC value from a predefined set of candidate values $\left\{\lambda_{1},\ldots ,\lambda_{d}\right\}$. The other tuning parameter *q* plays a pivotal role in determining the stringency of variable selection in TCVS. According to the thresholding criteria defined in Equation (6), opting for a lower value of *q* results in a more stringent threshold, typically yielding a smaller set of selected variables. Consequently, the choice of *q* is highly dependent on the specific objectives of the study and is left to the discretion of the researcher. In scenarios geared towards exploratory or discovery phases, where the allowance for false positives is somewhat greater, a higher *q* value may be chosen to facilitate preliminary findings. Conversely, during validation stages, where a stricter control over erroneous discoveries is desired, a lower *q* value would be more appropriate. For purpose of demonstration, we set *q* at 0.05 to exemplify the operational efficacy of TCVS across various numerical analyses conducted later in this article.

## 3 Results

### 3.1 Simulations

#### 3.1.1 Simulation setup

We first generated a count data matrix $W=(w_{ij})\in R^{n\times p}$ from a Dirichlet-Multinomial distribution, whose parameters were estimated from counts of 856 taxa in an upper-respiratory-tract microbiome dataset (Chen *et al.* 2012). To adjust for zero counts before CLR transformation, we added a pseudo-count of 0.5 to all counts before normalizing the count matrix **W** into the compositional matrix $X\in R^{n\times p}$ via $x_{ij}=w_{ij}/\sum_{l=1}^{p}w_{il}$. Simulations under various different settings and configurations were also conducted and reported in Section B of the online supplementary materials. Finally, we applied CLR transformation to **X** to obtain $Z\in R^{n\times p}$. We fixed the sample size at n=200 and varied the dimension of variables with p=60,300 and 856 to reflect different tree sizes. The response variable $y_{i}$ was then simulated from $y_{i}=\sum_{j=1}^{p}z_{ij}\beta_{j}+\epsilon_{i}$, where error terms $\epsilon_{i}$’s were independently and identically distributed as N(0,1). The true coefficient vector $\beta ={\left(\beta_{1},\ldots ,\beta_{p}\right)}^{⊤}$ was configured as follows:

- Setting I: $\beta_{1}=1,\;\beta_{2}=-1,\;\beta_{4}=0.8,\;\beta_{5}=-0.8,\;\beta_{8}=-1.5,\;\beta_{9}=-0.5,\;\beta_{10}=2,\;\beta_{11}=1.2,\;\beta_{12}=-1.2,\;\beta_{18}=0.7,\;\beta_{19}=0.8,\;\beta_{20}=-1.5$, $\beta_{j}=0$ otherwise.
- Setting II: $\beta_{1}=-0.5,\;\beta_{8}=2,\;\beta_{16}=-1.3,\;\beta_{26}=0.5,\;\beta_{29}=1.3,\;\beta_{34}=-2,\;\beta_{41}=0.8,\;\beta_{44}=-0.4,\;\beta_{47}=-1.6,\;\beta_{49}=-1.3,\;\beta_{51}=1.3,\;\beta_{53}=1.2$, $\beta_{j}=0$ otherwise.

**Figure 1.**
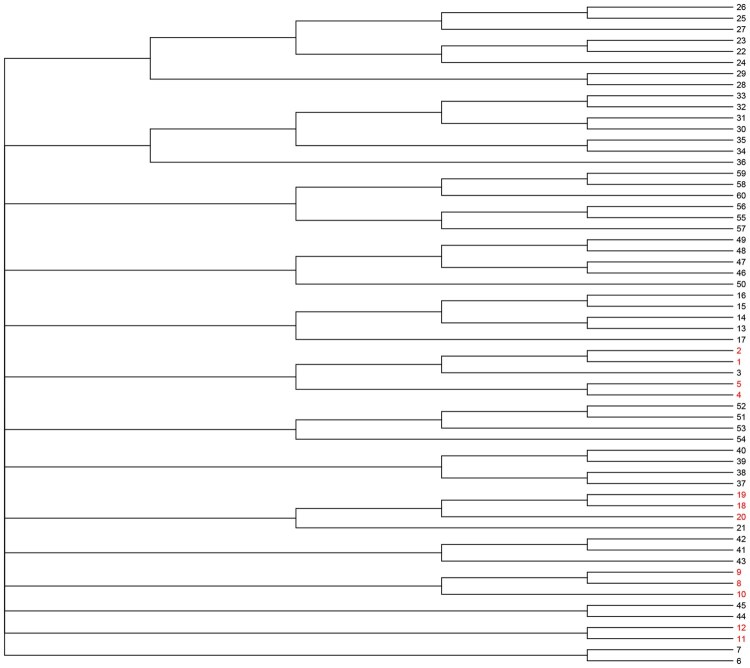
Tree structure used in simulation Setting I, where taxa with $\beta_{j}\neq 0$ are marked in red.

In our analysis, we began by examining a taxonomic tree *T* featuring p=60 terminal nodes, as illustrated in Figure 1. For Setting I, the taxa set {1,2,4,5,8,9,10,11,12,18,19,20} encompasses all true signals, aligning with the tree’s structural configuration and its internal nodes as depicted in Figure 1. Conversely, in Setting II, the true signal distribution deviates from the tree’s inherent structure, with true signals being dispersed randomly across the tree (illustrated in Figure S1 of the online supplementary materials). This experimental design was extended to taxonomic trees *T* with p=300 and p=856 nodes, where Setting I adheres to the presupposition that signal locations are relatively clustered and concentrated on the tree, while Setting II challenges this assumption by scattering signals randomly throughout the tree. Notably, irrespective of the dimension *p*, the count of non-zero coefficients remained constant at 12 in both settings. This setup allowed us to investigate the robustness of TCVS against varying degrees of background noise, essentially by altering the dimensionality of the compositional covariates.

Upon generating the dataset, we implemented TCVS and compared it with the compositional Lasso method (Lin *et al.* 2014) (henceforth denoted as CLasso) and the TASSO method (Wang *et al.* 2017). Incorporating auxiliary noise copies and the tree structure are the two factors that distinguish TCVS from CLasso. To evaluate the benefit of incorporating tree structure in TCVS, we evaluated an approach that resolves the optimization problem denoted by Equation (3), which we refer to as TLasso for simplicity. Similarly, to assess the benefit of incorporating auxiliary noise copies as a countermeasure against potential noise sources, we evaluated a method that applies Knockoff copies to the CLasso regression model. To distinguish it from a previous two-stage method (Srinivasan *et al.* 2021), we termed this intermediate method as CLassoKF hereafter. Also, for a fair comparison with TCVS, the model-X knockoff copies (Candes *et al.* 2018) were used in CLassoKF. Finally, it’s important to note that TASSO incorporates a ridge penalty on the coefficients of leaf nodes, which enables it to select subcompositions or internal nodes but does not facilitate sparsity among leaf nodes (Wang *et al.* 2017, Liu *et al.* 2022). In order to facilitate a comparison between TASSO and the other methods, it was necessary to apply hard thresholding to the TASSO estimates ${\hat{\beta }}_{j}(j=1,\ldots ,p)$, setting small coefficients below a certain threshold to zero. We employed three distinct threshold values $c=10^{-2},10^{-4},10^{-6}$ and manually truncated TASSO coefficients below these thresholds to zero. Results for TASSO with $c=10^{-2}$ are discussed in the main text, while outcomes for other thresholds are similar and hence are reported in Section B.1 of the online Supplementary Materials. Tuning parameters for all competing methods, including CLasso, TLasso, CLassoKF, and TASSO, were selected using the Bayesian Information Criterion (BIC), consistent with the approach used for TCVS.

To compare different methods, we calculated the True Positive Rate (TPR) and False Positive Rate (FPR), as:


$$\mathrm{TPR}=\frac{\#\{{\hat{\beta }}_{j}\neq 0\cap \beta_{j}^{0}\neq 0\}}{\#\{\beta_{j}^{0}\neq 0\}},\mathrm{FPR}=\frac{\#\{{\hat{\beta }}_{j}\neq 0\cap \beta_{j}^{0}=0\}}{\#\{\beta_{j}^{0}=0\}},$$

where $\hat{\beta }$ and $\beta^{0}$ represent the estimated and true regression coefficients, respectively. The averages of TPR and FPR are computed over 100 simulation replicates. Computational costs of different methods were also compared in Section B.6 of the online supplementary materials.

#### 3.1.2 Simulation results

The comparative analysis of selection performance among different methods is detailed in Table 1. Under Setting I, TLasso outperformed CLasso in terms of FPR, a result anticipated due to the tree-related signal associations effectively captured by TLasso. Conversely, in scenarios where the true coefficients did not align with the tree structure, the penalty delineated in Equation (3) becomes misaligned, narrowing the performance gap between TLasso and CLasso under Setting II compared to that under Setting I. The same argument applies to TCVS, which has a much better performance under Setting I compared to Setting II. The introduction of auxiliary noisy contrasts in TCVS and CLassoKF significantly enhances FPR outcomes across all tested scenarios, outperforming TLasso and CLasso. Finally, TASSO’s implementation of ridge penalties on leaf nodes precludes it from achieving model parsimony for leaf nodes, resulting in elevated FPRs across all settings, even under the most stringent false discovery threshold of $10^{-2}$. TASSO’s performance under alternative thresholds exhibits similar trends and is documented in Table S1 within the Supplementary Materials online.

**Table 1.** Simulation results of TCVS, TLasso, CLassoKF, CLasso and TASSO under different settings.

		Setting I		Setting II	
*p*	Method	TPR	FPR	TPR	FPR
60	TCVS	98.6%	3.7%	92.6%	6.7%
	TLasso	99.3%	7.1%	91.6%	31.7%
	CLassoKF	84.9%	1.2%	78.0%	1.3%
	CLasso	99.8%	27.7%	98.8%	28.3%
	TASSO	100.0%	16.6%	99.1%	76.9%
300	TCVS	97.0%	0.4%	86.6%	0.4%
	TLasso	98.3%	1.1%	67.5%	4.0%
	CLassoKF	90.0%	0.4%	79.4%	0.5%
	CLasso	99.3%	9.2%	98.0%	9.3%
	TASSO	59.7%	79.8%	61.8%	82.9%
856	TCVS	96.3%	0.1%	80.6%	0.2%
	TLasso	96.6%	0.5%	49.3%	1.1%
	CLassoKF	83.3%	0.4%	73.5%	0.4%
	CLasso	98.7%	3.9%	97.1%	4.4%
	TASSO	71.3%	49.8%	60.8%	52.8%

As noise levels escalate (p=300 and 856), a notable trend emerges: all methods except for TASSO exhibit reduced FPRs (especially for TLasso and CLasso). This phenomenon largely stems from the “denominator effect” within the FPR calculation, where the increase in true negatives with rising *p* values (given the constant number of non-zero coefficients) leads to lower FPRs. The overarching conclusions drawn from the low-dimensional case (p=60) largely persist across higher dimensions. TASSO consistently fails to yield a sparse model, resulting in the highest FPR. TLasso and CLasso display competitive selection efficacy, with their relative performance hinging on the fulfillment of underlying assumptions. CLassoKF tends to improve the FPR performance of CLasso by sacrificing the TPR a little bit. Ultimately, by incorporating both tree structure and auxiliary noise copies, TCVS consistently demonstrates superior variable selection capabilities relative to all other methods in both settings examined.

A potential concern of our method is selecting weaker signals since these signals are further diluted by additional auxiliary noises introduced in TCVS. While we set magnitudes of non-zero true regression coefficients in Settings I and II by taking those used in CLasso (Lin *et al.* 2014) and TASSO (Wang *et al.* 2017), it would be beneficial to provide a more comprehensive understanding of TCVS’s performance under weaker effect sizes. To this end, we conducted additional simulation studies with smaller regression coefficients $\beta_{j}$’s. Specifically, we choose nine equally spaced points from 0.2 to 1 as the shrinkage factor multiplied by the previous coefficient vectors used in Settings I and II as our new regression coefficients. To synthesize TPR and FPR into a more comprehensive performance metric, we calculated the F-score, defined as $F=2{\left(1/P+1/R\right)}^{-1}$, serving to balance precision (P)—the proportion of correctly identified non-zero coefficients among all identified non-zero coefficients—and recall (R), essentially the TPR.

**Figure 2.**
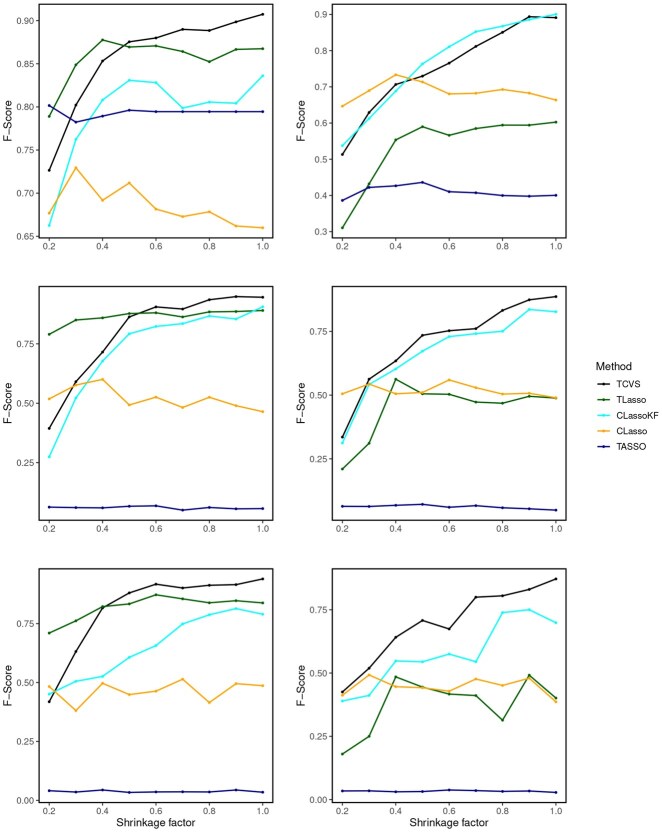
F-scores of different methods with different effect sizes. The left panel corresponds to Setting I and the right panel corresponds to Setting II. The top, middle and bottom row displays the scenario of p=60, 300, and 856, respectively.

The F-scores are averaged over 20 simulation replicates per effect size shrinkage factor and then displayed in Figure 2 across different shrinkage factors. When the effect size is relatively strong, TCVS consistently outperforms its counterparts by maintaining the highest F-scores across different scenarios. However, results in Figure 2 also reveal a potential limitation of TCVS: the introduction of additional noise by TCVS may not always be advantageous, particularly when dealing with very small effect sizes. Under Setting I, the TLasso method has the second best performance, and CLassoKF is the second best one under Setting II, which are all as expected and well aligned with our simulation results observed in Table 1. While TCVS demonstrates enhanced signal selection accuracy over existing methods in scenarios with moderate to strong signals, its efficacy diminishes as signal strength wanes. Finally, when the underlying association pattern shifts from Setting I and Setting II, both tree-based methods (TCVS and TLasso) suffer from loss of efficiency. The loss of efficiency in TLasso is much larger than that of TCVS, which might be because TCVS still enjoys efficiency gain brought by its auxiliary noises component. Overall, TCVS tends to be the best method or close to the best method, no matter whether the tree structure is consistent with the underlying pattern of association signals, as long as these signals are not too weak.

### 3.2 Real data analysis

To showcase the applicability of our approach, we employed TCVS, alongside TLasso, CLassoKF and CLasso, on a dataset from a gut microbiome study (Wu *et al.* 2011), made available through R package miLineage (Tang *et al.* 2017). The TASSO method assumes sparsity at subcompositions (i.e., internal nodes of the taxonomic tree), which is very different from the other four methods and can lead to a very different performance as observed in the previous simulations section. Hence, the model designed for TCVS, TLasso, CLassoKF and CLasso to analyze this dataset may not be appropriate for TASSO and hence we omit TASSO in our real data analysis. This gut microbiome dataset comprises an OTU table with n=96 samples and p=80 taxa, in addition to a taxonomy classification for these 80 taxa. This classification facilitates construction of a tree structure (Figure S2 in the online supplementary materials). The dataset also includes measurements of Body Mass Index (BMI), total fat intake and total caloric intake for each participant, with our analysis focusing on identifying BMI-associated gut microbial taxa after adjusting for effects of total fat and caloric intake. We transformed the count data into a compositional format by first adding a pseudo count of 0.5 to the original sequencing counts, followed by employing the centered log-ratio transformation to obtain our regression design matrix $Z\in R^{96\times 80}$. We applied the TCVS method directly to the transformed data and implemented other methods for comparison. The tuning of parameters in each method was selected by the same procedure used in previous simulation studies.

To achieve robust selection outcomes, we employed resampling techniques to generate 100 bootstrap samples of size n/2 for model training, with the remainder used for evaluation. Let $\left(y^{\left(1\right)},Z^{\left(1\right)}\right)$ represent the training dataset and $\left(y^{\left(2\right)},Z^{\left(2\right)}\right)$ be the evaluation dataset. When applying TLasso, CLasso, and TASSO to $\left(y^{\left(1\right)},Z^{\left(1\right)}\right)$, we obtain an estimated coefficient vector ${\hat{\beta }}^{\left(1\right)}$, identifying the set of selected variables. This set enables the evaluation of model predictive performance on $\left(y^{\left(2\right)},Z^{\left(2\right)}\right)$ by computing the mean squared prediction error (MSPE) as $||y^{\left(2\right)}-Z^{\left(2\right)}{\hat{\beta }}^{\left(1\right)}|{|}_{2}^{2}/n_{2}$, where $n_{2}$ is the size of dataset $\left(y^{\left(2\right)},Z^{\left(2\right)}\right)$. This approach for TCVS and CLassoKF are more intricate due to their reliance on regression coefficients from an augmented model, making direct comparisons with TLasso, CLasso, and TASSO challenging. To facilitate a meaningful comparison, we denote the set of variables selected by TCVS (or CLassoKF) using $\left(y^{\left(1\right)},Z^{\left(1\right)}\right)$ as *S*, and $y_{S}$ and $Z_{S}$ as the corresponding subvector or submatrix of indices in *S*. The evaluation dataset $\left(y^{\left(2\right)},Z^{\left(2\right)}\right)$ is split into two parts: 80% for fitting a regression model and 20% for assessing the MSPE. Let $\left(y^{\left(21\right)},Z^{\left(21\right)}\right)$ and $\left(y^{\left(22\right)},Z^{\left(22\right)}\right)$ represent these two subsets, respectively. Regression coefficients are then estimated by solving ${\hat{\beta }}_{S}^{\left(2\right)}=\underset{\beta_{S}}{\;\text{arg}\;\text{min}\;}\frac{1}{2n_{21}}||y_{S}^{\left(21\right)}-Z_{S}^{\left(21\right)}\beta_{S}|{|}_{2}^{2},\;s.t.,\sum_{j\in S}\beta_{Sj}=0,$ with the MSPE subsequently calculated as $||y^{\left(22\right)}-Z_{S}^{\left(22\right)}{\hat{\beta }}_{S}^{\left(2\right)}|{|}_{2}^{2}/n_{22}$.

In assessing the variable selection outcomes of different methods within the resampling framework described above, we focused on taxa selected in at least 10% of the 100 resampling iterations. The number of taxa selected by TCVS, TLasso, CLassoKF and CLasso are 4, 60, 2, and 21, respectively. TCVS identified a comparatively smaller number of signals, consistent with expectations set by prior simulation studies. Table 1 highlights that the FPR for TCVS and CLassoKF is markedly lower than the other three methods under p=60, coinciding with the variations noted in the number of findings for different methods. This might suggest that the heightened FPR associated with TLasso and CLasso could lead to unnecessary and labor-intensive validation efforts in subsequent biological investigations, and the conservative selection results of TCVS and CLassoKF might be advantageous, particularly when prioritizing the credibility of statistical findings. Notably, the four genera identified by TCVS are *Acidaminococcus, Alistipes, Allisonella* and *Oscillibacter*, where *Alistipes* belongs to the Bacteroidetes phylum and the other three belong to the Firmicutes phylum. It has been widely known that the Firmicutes/Bacteroidetes (F/B) ratio is associated with the BMI (Wu *et al.* 2011). Our findings might also support this conclusion. Finally, among these four genera, *Alistipes* and *Oscillibacter* are also detected by all of the other methods, further attesting to the reliability of TCVS. Moreover, we evaluated the predictive accuracy of models generated by the various methods. The mean prediction errors, averaged over 100 replications, for TCVS, TLasso, CLassoKF and CLasso were 29.04, 43.10, 30.48 and 30.79, respectively, with corresponding standard errors of 0.16, 0.22, 0.09 and 0.16. The predictive performance of TCVS, CLassoKF, and CLasso significantly outperforms that of TLasso, showcasing their superiority in this dataset. To conclude, the TCVS methodology not only ensures the most accurate variable selection but also achieves the highest model prediction accuracy in the analysis of gut microbiome data.

## 4 Discussion

In this work, we have introduced the TCVS method, designed to identify outcome-associated components within high-dimensional microbial compositional data. TCVS uniquely incorporates hierarchical taxonomic tree information inherent among microbial taxa to enhance the variable selection process. A distinctive aspect of TCVS is the integration of auxiliary noise variables into the selection mechanism, aiming to refine the algorithm towards more precise outcomes. The construction of these informative noise variables is critical for enhancing selection accuracy and the strategy of augmenting statistical variable selection with auxiliary noises proves to be advantageous, particularly when the signal strength exceeds a minimal threshold. Through simulation studies, TCVS demonstrated exceptional accuracy, achieving near-perfect true positive rates and significantly low false positive rates in variable selection. Compared to existing methods that do not leverage auxiliary noises, TCVS’s performance is notably superior and remains consistent across various signal strength scenarios. Furthermore, the application of TCVS to real-world gut microbiome data further underscores its effectiveness and the practical value it offers. The results affirm the method’s capacity to deliver highly reliable variable selection and underscore the benefit of integrating auxiliary noises into the selection process for microbial compositional data analysis.

TCVS employs a strategic penalization method to pinpoint specific microbial attributes from an extensive pool of potential predictors. This is accomplished by applying an $L_{1}$ penalty to the leaf nodes and a tree-based group penalty, both of which are governed by a shared tuning parameter, λ, mirroring the approach used in the TASSO method where both penalties are equally weighted (Wang *et al.* 2017). Although employing distinct tuning parameters, $\lambda_{1}$ and $\lambda_{2}$, for each penalty could potentially enhance selection accuracy, this adjustment could significantly escalate the computational demands involved in identifying the optimal set of parameters across a two-dimensional grid.

The construction of knockoff variables utilizes the sequential conditional independent pairs algorithm (Candes *et al.* 2018), which necessitates the conditional distribution density functions for generating knockoff samples. This process is straightforward for multivariate normal distributions, prompting the use of the CLR transformation in TCVS to approximate the data distribution to multivariate normal. This approximation generally works for most settings considered in this article. When the joint distribution clearly departs from multivariate normal, the generation of knockoffs for more complex distributions remains a vibrant area of exploration, some alternative strategies based on deep generative models (Romano *et al.* 2020) or generative adversarial networks (Jordon *et al.* 2018) may be more appropriate for handling arbitrary and unspecified data distributions that are clearly non-Gaussian.

## Supplementary Material

btaf617_Supplementary_Data

## Data Availability

The gut microbiome data analyzed in this paper can be accessed from the R package miLineage. R code for implementing our method is available at https://github.com/Yicong1225/TCVS.
