# Supplementary material for: TCVS: tree-guided compositional variable selection analysis of microbiome data
Source: Bioinformatics. 2025 Nov 9;41(11):btaf617. doi: 10.1093/bioinformatics/btaf617 (PMC12629236; doi:10.1093/bioinformatics/btaf617)
Supplement: btaf617_Supplementary_Data [file btaf617_supplementary_data.pdf]

Supplementary Materials to "TCVS: Tree-guided compositional variable selection analysis of microbiome data"

Abstract

This supporting information file consists of three parts: (A) Additional figures demonstrating two tree structures used in simulation studies and real data analysis of the main text; (B) Additional simulation studies and results to illustrate the robustness of the proposed method; (C) Another case study example to demonstrate potential usefulness of our method to analyze real-world datasets.

A Additional figures

We first present the tree structure used in simulation Setting II of the main text when $p = 60$ is shown in Figure S1. Compared to the tree structure displayed in Figure 1 of the main text, true signals (shown in red) are less adjacent to each other on the tree. Then, the tree structure extracted from the taxonomy information of the miLineage data used in the real data analysis section of the main text is displayed in Figure S2.

B Additional simulation studies

B.1 Additional TASSO results

Let $(\hat{\beta}_1^r, \ldots, \hat{\beta}_p^r)$ be the raw estimated regression coefficients by the TASSO method. Due to the ridge penalty employed to leaf nodes, these TASSO estimates are not sparse as observed in the main text. To make a meaningful comparison with the other methods presented in the main text, we used some thresholding techniques to shrink small TASSO estimates to zeroes. In particular, we adopted the hard thresholding to calculate

$$\hat{\beta}_j = \hat{\beta}_j^r \cdot I[|\hat{\beta}_j^r| > c],$$

where $I[\cdot]$ is the indicator function and $c > 0$ is a certain cutoff value. We treated $\hat{\beta}_j$'s as our new TASSO estimates to be compared with other methods. In the main text, we have used the cutoff value of $c = 0.01$. In this supplementary materials file, We present TASSO results with different cutoff values $c = 10^{-2}, 10^{-4}$ and 10^{-6} in Table S1 under the logistic-normal distribution setting described in the Section B.2 of this supplementary materials file.

As the cutoff value decreases, the TASSO estimates would be less sparse, and hence both TPR and FPR will tend to be larger. Under $p = 60$, the FPRs of TASSO-4 and TASSO-6 are much less favorable than those of TASSO-2, while TPRs of the three methods are all saturated at 100%. When $p = 300$ or $p = 1000$, the results of three methods are similar. Overall, the choice of cutoff value c does not have a huge impact on the performance of TASSO under both settings considered in our simulations. For this reason, we pick TASSO with $c = 0.01$ as a representative to be compared with other methods in numerical studies presented in the remaining of this article.

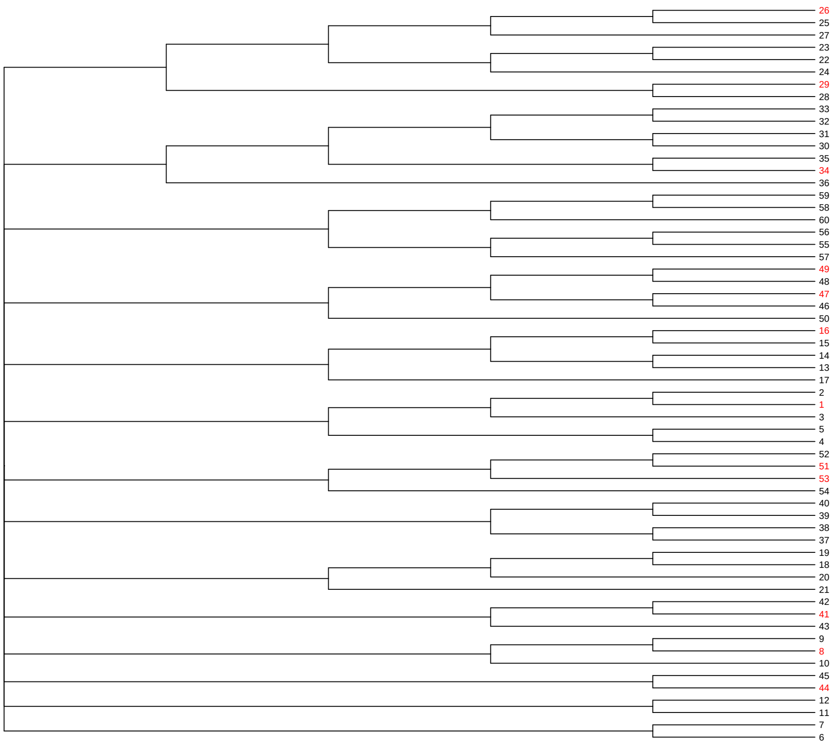

Figure S1: Tree structure used in Setting II, where taxa with $\beta_j \neq 0$ are marked in red.

B.2 Simulations under the logistic-normal distribution

In simulations conducted in the main text, Dirichlet-Multinomial counts were first simulated and then transformed into compositions. In some scenarios, such count variables may be absent and only compositions are available. One popular strategy to directly generate these compositions is to use the logistic-normal distribution as done in previous publications (Lin et al., 2014; Wang et al., 2017). In this section, we followed the designs used in TASSO (Wang et al., 2017) and the compositional Lasso (Lin et al., 2014) to generate the simulated data. Initially, we generated a data matrix $W = (w_{ij}) \in \mathbb{R}^{n\times p}$ from a multivariate normal distribution, characterized by $\mu = (\mu_1, ..., \mu_p)^\top \in \mathbb{R}^p$ and $\Sigma = (\Sigma_{ij}) \in \mathbb{R}^{p\times p}$, where the mean vector was specified as the zero vector and the covariance matrix was defined by $\Sigma_{ij} = \rho^{|i-j|}$ with $\rho = 0.5$ for all $i, j = 1, \dots, p$. To generate the compositional covariate matrix $\mathbf{X} \in \mathbb{R}^{n\times p}$, we applied the transformation $x_{ij} = \frac{\exp(w_{ij})}{\sum_{l=1}^{p} \exp(w_{il})}$ and the CLR transformation yielded the matrix $\mathbf{Z} \in \mathbb{R}^{n\times p}$ through the formula $z_{ij} = \log(x_{ij}) - \frac{1}{p}\sum_{l=1}^{p} \log(x_{il})$, for $i = 1, \dots, n$ and $j = 1, \dots, p$. After generating the design matrix $\mathbf{Z}$, the remaining simulation setups were the same as those described in the main text. The performance of different methods under this new logistic normal distribution are reported in Table S2. Then, as done in the main text, we varied the effect sizes of the original regression model by multiplying a shrinkage factor to the original regression coefficient vector and then present the F-scores of different methods in Figure S3. Results shown in Table S2 are close to those obtained under the Dirichlet-multinomial distribution presented in the main text (i.e., Table 1 of the main text). Similar to what we have observed in Figure 2 of the main text, based on Figure S3, TCVS tends to be the best methods or close to the best method under both settings as long as the effect sizes of signals are not too small.

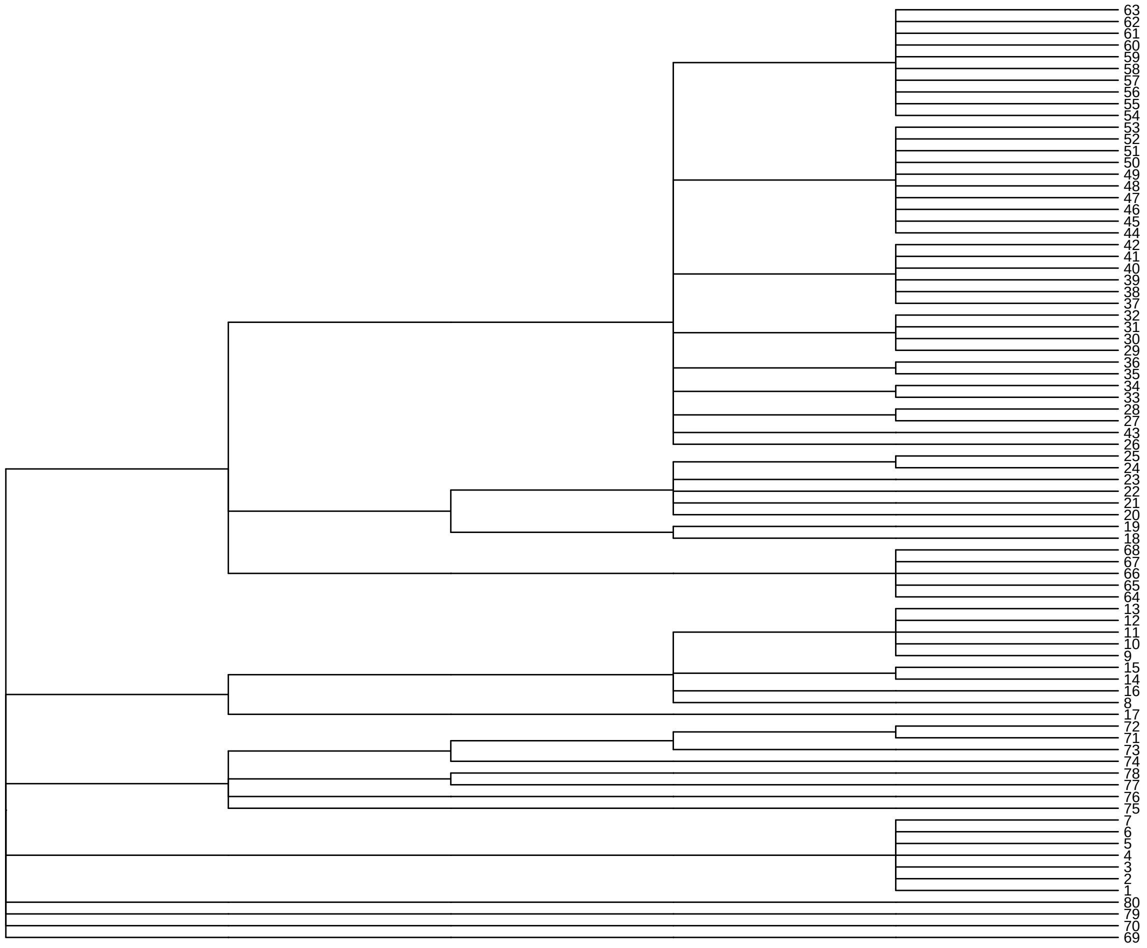

Figure S2: Tree structure for the `miLineage` data.

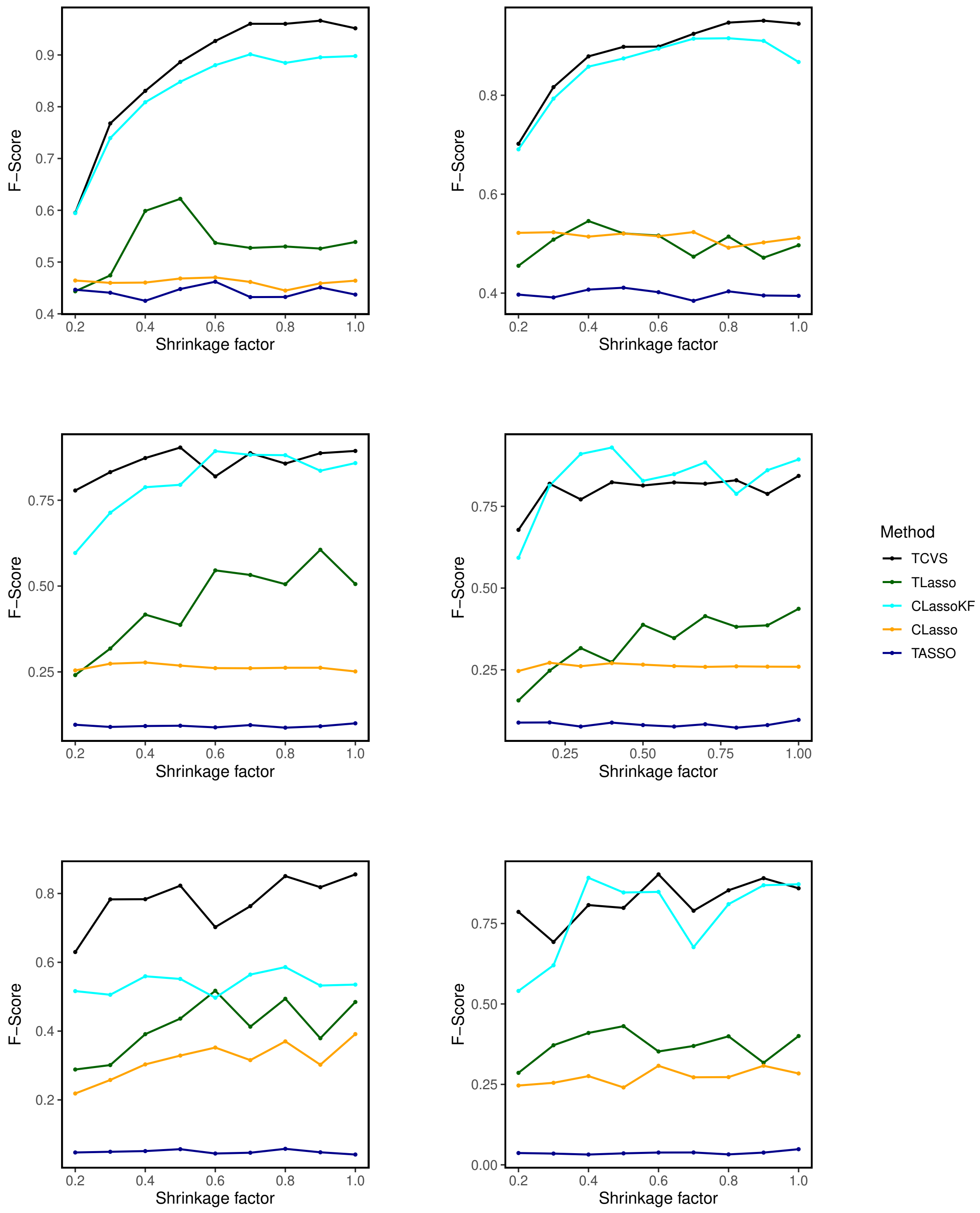

Figure S3: F-scores of different methods with different effect sizes under the logistic-normal distribution. The left panel corresponds to Setting I and the right panel corresponds to Setting II. The top, middle and bottom row displays the scenario of $p = 60$, 300, and 1000, respectively.

Table S1: Simulation results of TASSO with different thresholds under different settings. TASSO-2 denotes TASSO with a threshold of 10^{-2}, TASSO-4 denotes TASSO with a threshold of 10^{-4} and TASSO-6 denotes TASSO with a threshold of 10^{-6}.

		Setting I		Setting II	
p	Method	TPR	FPR	TPR	FPR
60	TASSO-2	100.0%	64.2%	100.0%	75.2%
	TASSO-4	100.0%	79.1%	100.0%	90.4%
	TASSO-6	100.0%	79.3%	100.0%	90.5%
300	TASSO-2	73.3%	60.4%	66.5%	59.6%
	TASSO-4	74.5%	62.7%	67.1%	60.8%
	TASSO-6	76.8%	64.2%	68.7%	61.7%
1000	TASSO-2	66.3%	34.0%	50.9%	34.8%
	TASSO-4	66.5%	36.8%	51.8%	36.5%
	TASSO-6	68.7%	38.9%	52.8%	37.4%

Table S2: Simulation results of different methods under the logistic normal distribution setting.

		Setting I		Setting II	
p	Method	TPR	FPR	TPR	FPR
60	TCVS	99.8%	2.8%	98.5%	3.3%
	TLasso	100.0%	49.0%	100.0%	48.9%
	CLassoKF	91.7%	2.5%	90.6%	3.3%
	CLasso	100.0%	58.5%	100.0%	48.1%
	TASSO	100.0%	64.2%	100.0%	75.2%
300	TCVS	100.0%	1.6%	100.0%	2.2%
	TLasso	98.1%	11.2%	94.9%	19.3%
	CLassoKF	86.1%	0.5%	86.0%	0.3%
	CLasso	100.0%	23.4%	100.0%	23.7%
	TASSO	73.3%	60.4%	66.5%	59.6%
1000	TCVS	96.8%	0.1%	96.3%	0.5%
	TLasso	96.9%	2.8%	90.6%	5.2%
	CLassoKF	82.3%	0.1%	87.2%	0.1%
	CLasso	79.3%	3.4%	100.0%	6.5%
	TASSO	66.3%	34.0%	50.9%	34.8%

B.3 Sensitivity analysis for different zero replacement strategies

In the main text, a pseudo count of 0.5 was added to the original counts prior to downstream statistical analysis (i.e., CLR transformation). This value was suggested in a previous publication (Shi et al., 2022), which has shown its optimality under certain mild conditions. To see how this value could affect the performance of different methods, we conducted additional simulations by introducing a series of different pseudo-counts to each sample's count data prior to normalizing the simulated counts into compositions and then CLRs, as described in the main text. New results under $p = 60$ are presented in Table S3. Results under other dimensions are similar and hence not reported. As can be seen in Table S3, most methods tend to have a better TPR with a smaller pseudo count value and the only exception is CLasso, whose TPR increases with the pseudo count value. On the other hand, this pseudo-count effect on FPR seems to be opposite and most methods tends to have a slightly better FPR with increasing pseudo-count values. Overall, the impact of different pseudo count values are relatively weak and can not change the conclusions made in the main text about comparisons between different methods.

Table S3: Performance of different methods with different pseudo-count values for zero replacement under $p = 60$.

		Setting I		Setting II	
Pseudo count	Method	TPR	FPR	TPR	FPR
0.001	TCVS	100.0%	3.3%	99.8%	6.9%
	TLasso	100.0%	7.7%	99.7%	48.6%
	CLassoKF	97.1%	2.2%	92.9%	1.9%
	CLasso	66.0%	23.9%	85.0%	24.0%
	TASSO	100.0%	9.3%	99.8%	64.6%
0.01	TCVS	100.0%	3.6%	99.4%	6.5%
	TLasso	100.0%	7.4%	99.3%	43.5%
	CLassoKF	96.2%	2.2%	92.8%	1.7%
	CLasso	77.0%	22.3%	98.0%	28.3%
	TASSO	100.0%	12.3%	99.9%	69.6%
0.1	TCVS	100.0%	4.2%	97.5%	7.3%
	TLasso	100.0%	7.0%	97.0%	38.8%
	CLassoKF	95.5%	2.1%	85.9%	1.6%
	CLasso	93.0%	25.8%	99.8%	28.9%
	TASSO	100.0%	15.1%	99.8%	75.3%
0.5	TCVS	98.6%	3.7%	92.6%	6.7%
	TLasso	99.3%	7.1%	91.6%	31.7%
	CLassoKF	84.9%	1.2%	78.0%	1.3%
	CLasso	99.8%	27.7%	98.8%	28.3%
	TASSO	100.0%	16.6%	99.1%	76.9%
1	TCVS	97.9%	3.4%	88.5%	6.6%
	TLasso	98.5%	7.1%	87.2%	28.8%
	CLassoKF	85.0%	1.6%	71.4%	1.1%
	CLasso	99.4%	26.5%	97.8%	27.3%
	TASSO	100.0%	16.1%	98.3%	76.9%

B.4 Sensitivity analysis for different normalization strategies

In simulations conducted in the main text, we first used the Dirichlet-multinomial distribution to generate an OTU count table and then counts in each column is scaled by the column's sum to obtain proportions before further analysis. There are various normalization methods to pre-process microbiome data before downstream computational and statistical analysis (Weiss et al., 2017) and selecting an appropriate one depends on both data characteristics and also the goal of analysis. One remarkable feature of TCVS analysis is the compositional nature in regression predictors, and a particular normalization method to fit this goal is rarefaction. That is, each column of the OTU table is subsampled to even depth without replacement. To this end, we conducted additional numerical simulations to compare whether rarefaction and proportion have impacts on performance of different methods. Results under $p = 60$ are presented in Table S4. When changing the normalization method from rarefaction to proportion, it seems that all methods have a much better TPR only by sacrificing a little bit of FPR, which is slightly different from what we have observed in Table S3. However, like what we have observed in in Table S3, pairwise comparison between different methods are similar. Under both normalization methods, TCVS has the second best FPR, which is slightly larger than that of CLassoKF but are much smaller than the others. However, the TPR of TCVS is always the highest or second highest, and is much better than that of CLassoKF. The overall performance of TCVS is always the best, especially under Setting I.

Table S4: Performance of different methods with different normalization methods $p = 60$ based on 100 replicates.

		Setting I		Setting II	
Normalization	Method	TPR	FPR	TPR	FPR
Rarefaction	TCVS	92.6%	3.4%	73.8%	7.3%
	TLasso	92.3%	6.0%	62.3%	17.7%
	CLassoKF	74.8%	1.5%	47.7%	1.4%
	CLasso	68.8%	18.2%	67.3%	17.3%
	TASSO	98.4%	17.0%	92.8%	70.6%
Proportion	TCVS	98.6%	3.7%	92.6%	6.7%
	TLasso	99.3%	7.1%	91.6%	31.7%
	CLassoKF	84.9%	1.2%	78.0%	1.3%
	CLasso	99.8%	27.7%	98.8%	28.3%
	TASSO	100.0%	16.6%	99.1%	76.9%

B.5 Sensitivity analysis for different taxonomy tree structures

B.5.1 Tree structure derived directly from real data

In simulation studies presented in the main text, we have used the same tree structure in Figure 1 (Setting I) and Figure S1 (Setting II) to design our numerical studies. In this section, we have tried different taxonomy tree structures to further demonstrate the robustness of TCVS. In particular, we have picked taxonomic tree structures derived directly from dataset collected from real-world microbiome research. One is the gut microbiome dataset with $p = 80$ leaf nodes (displayed in Figure S2) and the other is a mucosal microbiome dataset with $p = 291$ leaf nodes (more details

are provided in Section C of this supplementary file). These tree structures are used to generate the membership vector of leaf nodes for grouping purpose in TCVS.

As we have done previously in the main text, we also designed two settings for these two new tree structures. Under Setting I, we carefully designed the taxa set to contain nonzero regression coefficients, in order to make sure that these true signals are aligned with the underlying tree structure. In particular, the true regression coefficients under two new tree structures were picked as:

- Setting I (p=80): $\beta_1 = 1,\ \beta_2 = -1,\ \beta_3 = 0.8,\ \beta_4 = -0.8,\ \beta_5 = -1.5,\ \beta_6 = -0.5,\ \beta_7 = 2,\ \beta_{14} = 0.7,\ \beta_{15} = 0.8,\ \beta_{16} = -1.5,\ \beta_{18} = 1.2,\ \beta_{19} = -1.2,\ \beta_j = 0$ otherwise.
- Setting I (p=291): $\beta_{115} = -1.5,\ \beta_{116} = 0.7,\ \beta_{117} = 0.8,\ \beta_{131} = 0.8,\ \beta_{132} = -0.8,\ \beta_{133} = -1,\ \beta_{134} = 1,\ \beta_{137} = 2,\ \beta_{138} = -1.5,\ \beta_{139} = -0.5,\ \beta_j = 0$ otherwise.

Under Setting II, these true signals were distributed in a manner that deviates from the underlying tree structure, being randomly scattered across the tree. In particular, we set true regression coefficients under both new tree structures as:

- Setting II (p=80 or p = 291): $\beta_1 = -0.5,\ \beta_8 = 2,\ \beta_{16} = -1.3,\ \beta_{26} = 0.5,\ \beta_{29} = 1.3,\ \beta_{34} = -2,\ \beta_{41} = 0.8,\ \beta_{44} = -0.4,\ \beta_{47} = -1.6,\ \beta_{49} = -1.3,\ \beta_{51} = 1.3,\ \beta_{53} = 1.2,\ \beta_j = 0$ otherwise.

Simulation results under these new tree structures are summarized in Table S5, which are quite consistent with those reported in Table 1 of the main text, which confirms that the performance of TCVS remains robust when using real taxonomic tree structures, thereby supporting the validity of our proposed method under more realistic conditions.

Table S5: Simulation results of different methods based on actual microbiome phylogenetic trees under the Dirichlet-Multinomial distribution.

		Setting I		Setting II	
p	Method	TPR	FPR	TPR	FPR
80	TCVS	93.8%	3.1%	89.2%	4.5%
	TLasso	89.4%	7.9%	91.0%	22.6%
	CLassoKF	79.1%	1.0%	69.8%	0.5%
	CLasso	99.3%	21.2%	98.8%	23.5%
	TASSO	99.0%	23.0%	97.9%	34.6%
291	TCVS	90.5%	0.5%	82.3%	0.4%
	TLasso	69.1%	4.4%	38.2%	4.4%
	CLassoKF	81.5%	0.5%	80.3%	0.5%
	CLasso	99.4%	8.2%	98.1%	9.5%
	TASSO	97.1%	78.1%	74.5%	77.8%

B.5.2 Misspecified tree structure

In the main text, we have designed Simulation Setting II (randomly scattered signals) to partially address signal–tree mismatch. As pointed out by a reviewer, a more challenging scenario would be the case that the tree itself is structurally misspecified (e.g., by randomly perturbing subtree

memberships). To this end, we have designed a new simulation setting in which the taxonomic tree structure is deliberately misspecified to test the performance of our method under such a scenario. Specifically, we first generate the data following the strategy described in Setting I (i.e., true signals concentrated on the tree). Then, we randomly permute a subset of leaf labels (i.e., a subset of the $\mathbf{m}_v$ vectors that encode subtree memberships) such that the tree after perturbation no longer matches the one generating the data. This setting enables a direct evaluation of the robustness of TCVS when the biological prior is biased. Results under dimensions of $p = 60$ and 300 were summarized in Table S6, which shows that the performance of TCVS remains similar to those reported in the main text, whereas the other tree-based method TLasso exhibits notable deterioration. These results further demonstrate that TCVS is robust to biases in the biological prior, thereby reinforcing the reliability of our method even under misspecified taxonomic structures.

Table S6: Additional simulation results comparing different methods under a misspecified tree structure.

	$p = 60$		$p = 300$	
Method	TPR	FPR	TPR	FPR
TCVS	97.0%	5.4%	94.0%	0.5%
TLasso	97.3%	15.7%	55.6%	2.8%
CLassoKF	88.6%	1.3%	92.1%	0.5%
CLasso	98.9%	26.6%	99.6%	10.1%
TASSO	99.9%	38.9%	83.3%	86.1%

B.6 Comparison of computational cost of different methods

Computational cost is critical in practice. In this section, we compare the computing time of each method using simulated datasets. Table S7 presents the computing time (in seconds) for different methods under varying dimensional settings, reported as the mean (standard deviation) across 100 simulation replicates. The reported runtime of TCVS was obtained using 32 cores, demonstrating that the proposed method is highly *parallelizable*. Given that modern computing environments—ranging from personal laptops to computing servers—are typically equipped with multi-core processors, this parallelizability makes TCVS practically feasible and scalable. Moreover, while TCVS achieves markedly superior performance, the associated increase in computational cost is only modest and remains well within a practically acceptable range.

B.7 Verification of the approximate multivariate normality of CLR-transformed covariates

Let $\mathbf{Z} \in \mathbb{R}^{n \times p}$ denote the design matrix of CLR-transformed covariates. The distribution assumption of multivariate normality is needed when implementing the Sequential Conditional Independent Pairs Algorithm of the model-X knockoff method (Candes et al., 2018) to generate valid knockoff copies $\tilde{\mathbf{Z}}$ of $\mathbf{Z}$. The multivariate normality assumption will largely facilitate computing of conditional distribution densities required in the Sequential Conditional Independent Pairs Algorithm. We first filter out rare taxa using the criterion in Hu et al. (2022): a taxon is retained only if it is present in at least 20% of the samples. This filtering step removes extremely sparse taxa that carry little information and could otherwise distort the estimation of the covariance structure. To

Table S7: Computing time (seconds) for different methods under varying dimensional settings, reported as mean (standard deviation) across 100 replicates.

p	Method	Setting I	Setting II
60	TCVS	13.14(0.07)	13.16(0.04)
	TLasso	4.16(0.02)	4.20(0.02)
	CLassoKF	1.51(0.00)	1.57(0.01)
	CLasso	11.14(0.46)	11.11(0.48)
	TASSO	0.10(0.00)	0.11(0.00)
300	TCVS	497.03(1.64)	496.36(2.72)
	TLasso	40.66(0.14)	40.93(0.21)
	CLassoKF	4.09(0.02)	4.18(0.03)
	CLasso	712.65(10.28)	708.69(10.48)
	TASSO	3.93(0.03)	3.94(0.03)
856	TCVS	9737.21(35.19)	9634.54(20.01)
	TLasso	549.77(1.71)	540.88(1.62)
	CLassoKF	12.21(0.12)	12.38(0.09)
	CLasso	6703.38(79.24)	6630.24(78.89)
	TASSO	87.86(0.64)	87.41(0.34)

evaluate the multivariate normality of $\mathbf{Z} = (z_1, z_2, \cdots, z_n)^\top$, we compute the squared Mahalanobis distances for each observation z_i as follows:

$$D_i^2 = (z_i - \hat{\mu})^\top \hat{\Sigma}^{-1}(z_i - \hat{\mu}),$$

where $\hat{\mu}$ and $\hat{\Sigma}$ are the sample mean vector and covariance matrix of $\mathbf{Z}$. Under the null hypothesis of multivariate normality, $D_i^2 \sim \chi_p^2$ approximately. We next evaluate the multivariate normality assumption for both the simulated data and real datasets analyzed in the main text. In particular, we construct QQ plots by plotting the ordered D_i^2 values against the theoretical quantiles of the χ_p^2 distribution.

QQ plots for typical datasets generated in our simulation study (with different dimensions) are reported in Figure S4, and those for two real datasets analyzed in the main text are shown in Figure S5. As can be seen in both figures, the points in all QQ plots from the simulation data align closely with the diagonal line $y = x$ (the red solid line), indicating that the CLR-transformed data in our simulation study satisfy the multivariate normality assumption well.

C Mucosal microbiome data analysis

In the main text, we have analyzed a gut microbiome dataset with 96 samples and 80 taxa. It is also of interest to evaluate the performance of our method using a real-world dataset with larger sizes (both in terms of samples and taxa). To this end, we applied our method to a mucosal microbiome dataset collected from inflammatory bowel disease (IBD) patients (Morgan et al., 2015). A total of 255 samples were collected and the original mucosal microbiome data contain 7000 OTUs, with many singletons that were only detected in a single sample. Based on their taxonomic information, these OTUs have been first aggregated into 303 distinct genus-level taxa. Then, 12 taxa had missing taxonomic rank information, which cannot be accurately placed in the taxonomic tree, and hence were removed from our analysis. We used taxonomic ranks of the remaining $p = 291$ genus-level taxa to construct the taxonomic tree for downstream TCVS analysis.

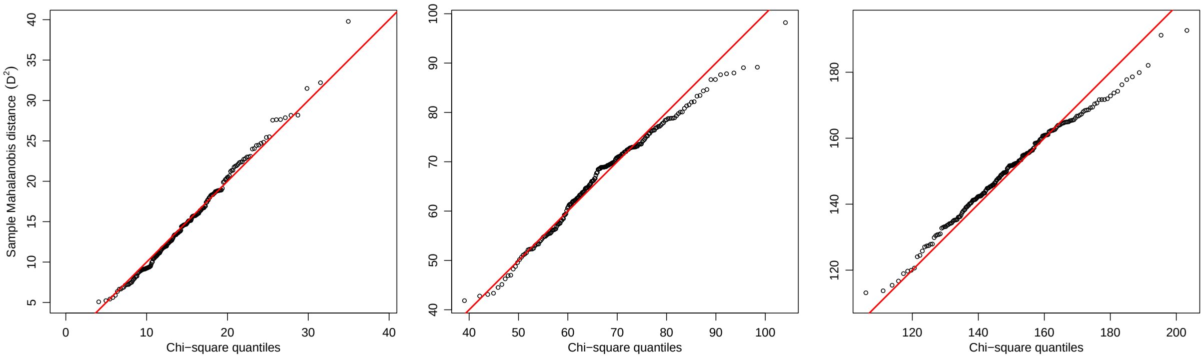

$p = 60$ $p = 300$ $p = 856$

Figure S4: QQ plots of simulated datasets

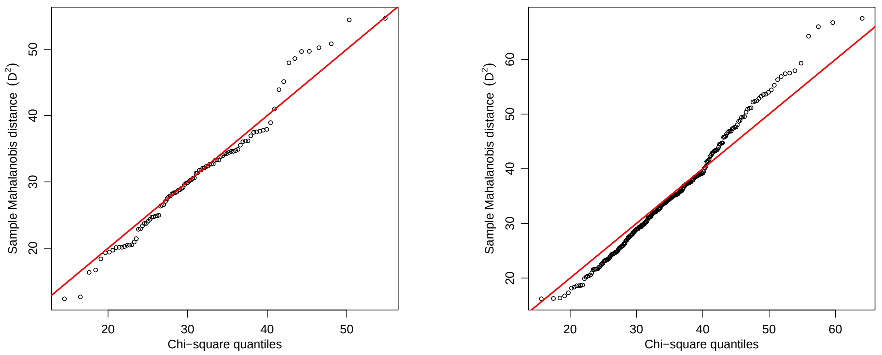

Gut microbiome dataset Mucosal microbiome dataset

Figure S5: QQ plot of the real datasets

Besides mucosal microbiome compositions, some clinical information was also measured and collected from these inflammatory bowel disease patients. A particular metric of interest is the inflammatory score measuring the severity of inflammation. Similar to the analysis of gut microbiome dataset presented in the main text, a log contrast regression model was used to study associations between inflammatory score and microbial taxa. In the real data analysis section presented in the main text, we have used bootstrap resampling to evaluate variable selection and prediction performance of different methods. In real-world datasets, it's common for small changes in the training data to lead to different variable selections. Hence, stability with respect to such perturbations is a desired property of a good variable selection method. To this end, we still use the bootstrap resampling strategy described in the main text to evaluate variable selection performance of different methods. The number of taxa selected by TCVS, TLasso, CLassoKF and CLasso are 2, 62, 1 and 31, respectively. Similar to the findings reported in the main text, TCVS tends to identify fewer signals, which likely represent true discoveries. Its conservative selection pattern, consistent with lower FPR, suggests that TCVS offers more credible results compared to the other methods. Notably, the two genera identified by TCVS are *Ruminococcus* and *Solibacillus*. Both have been linked to intestinal inflammation, with *Ruminococcus* implicated in inflammatory disorders of the

gut Kandasamy et al. (2023) and duodenal mucosa–associated *Solibacillus* observed in patients with intestinal mucosal alterations Gong et al. (2019). We next evaluated the predictive accuracy of models as described in the main text. The mean prediction errors, averaged over 100 replications, for TCVS, TLasso, CLassoKF and CLasso were 4.98, 5.70, 5.79 and 5.30, respectively, with corresponding standard errors of 0.26, 0.13, 0.25 and 0.08, respectively.

References

Candes, E., Y. Fan, L. Janson, and J. Lv (2018). Panning for gold:model-x knockoffs for high dimensional controlled variable selection. *Journal of the Royal Statistical Society: Series B (Statistical Methodology) 80*(3), 551–577.

Gong, J., L. Li, X. Zuo, and Y. Li (2019). Change of the duodenal mucosa-associated microbiota is related to intestinal metaplasia. *BMC microbiology 19*(1), 275.

Hu, Y., G. A. Satten, and Y.-J. Hu (2022). Locom: A logistic regression model for testing differential abundance in compositional microbiome data with false discovery rate control. *Proceedings of the National Academy of Sciences 119*(30), e2122788119.

Kandasamy, S., V. Letchumanan, K. W. Hong, K.-O. Chua, N. S. Ab Mutalib, A. L. O. Ng, L. C. Ming, H. X. Lim, S. Thurairajasingam, J. W.-F. Law, et al. (2023). The role of human gut microbe ruminococcus gnavus in inflammatory diseases. *Progress In Microbes & Molecular Biology 6*(1).

Lin, W., P. Shi, R. Feng, et al. (2014). Variable selection in regression with compositional covariates. *Biometrika 101*(4), 785–797.

Morgan, X. C., B. Kabakchiev, L. Waldron, A. D. Tyler, T. L. Tickle, R. Milgrom, J. M. Stempak, D. Gevers, R. J. Xavier, M. S. Silverberg, et al. (2015). Associations between host gene expression, the mucosal microbiome, and clinical outcome in the pelvic pouch of patients with inflammatory bowel disease. *Genome Biology 16*(1), 1–15.

Shi, P., Y. Zhou, and A. R. Zhang (2022). High-dimensional log-error-in-variable regression with applications to microbial compositional data analysis. *Biometrika 109*(2), 405–420.

Wang, T., H. Zhao, et al. (2017). Structured subcomposition selection in regression and its application to microbiome data analysis. *Annals of Applied Statistics 11*(2), 771–791.

Weiss, S., Z. Z. Xu, S. Peddada, A. Amir, K. Bittinger, A. Gonzalez, C. Lozupone, J. R. Zaneveld, Y. Vázquez-Baeza, A. Birmingham, et al. (2017). Normalization and microbial differential abundance strategies depend upon data characteristics. *Microbiome 5*(1), 27.
